# CDC Grand Rounds: Discovering New Diseases via Enhanced Partnership Between Public Health and Pathology Experts

**Published:** 2014-02-14

**Authors:** Sherif Zaki, Dianna M. Blau, James M. Hughes, Kurt B. Nolte, Ruth Lynfield, Wendy Carr, Tanja Popovic

**Affiliations:** 1 Infectious Diseases Pathology Branch, National Center for Emerging and Zoonotic Infectious Diseases, CDC; 2 Emory University School of Medicine, Atlanta, Georgia; 3 Office of the Medical Investigator, University of New Mexico School of Medicine; 4 Minnesota Department of Health; 5 Office of the Director, National Center for Emerging and Zoonotic Infectious Diseases, CDC; 6 Office of the Director, CDC

Despite advances in public health, medicine, and technology, infectious diseases remain a major source of illness and death worldwide. In the United States alone, unexplained deaths resulting from infectious disease agents have an estimated annual incidence of 0.5 per 100,000 persons aged 1–49 years (1). Emerging and newly recognized infections, such as hantavirus pulmonary syndrome and West Nile encephalitis, often are associated with life-threatening illnesses and death (Table 1). Other infectious diseases once thought to be on the decline, such as pertussis, again are becoming major public health threats. Animals increasingly are being recognized as potential vectors for infectious diseases affecting humans; approximately 75% of recently emerging human infectious diseases are of animal origin. Increasing global interconnectivity necessitates more rapid identification of infectious disease agents to prevent, treat, and control diseases.

Surveillance and rapid response for emerging infectious diseases remain cornerstones of CDC’s public health mission. There is a need for a holistic “One Health*” approach with interdisciplinary engagement, given the vital interconnectedness among humans, animals, and the environment. Fortunately, many partnerships, systems, and tools are available to use in pursuit of this goal. The strong public health partnership between CDC’s Infectious Diseases Pathology Branch and forensic pathologists and medical examiners, coupled with the use of state-of-the-art technologies, has facilitated explanation of many otherwise unexplained deaths, led to the discovery of new pathogens, and enabled the monitoring of unexplained deaths and critical illnesses at the state and local levels.

## The Pathologist and Public Health Partnership

Pathologists are among the first to encounter infectious disease outbreaks through their collaborative work with diverse specialists including epidemiologists, clinicians, veterinarians, and microbiologists, and are thus in an excellent position to discover emerging infectious diseases (2). Pathology has played a critical role in advancing the knowledge of emerging infectious diseases (3).

### Hantavirus at the Four Corners

For example, in 1993, an unexplained respiratory illness appeared in the Four Corners area (a region of the United States where the boundaries of Colorado, New Mexico, Arizona, and Utah meet) with reports of a influenza-like illness with high mortality rates in previously healthy young adults. The diligence of forensic pathologists in New Mexico in pursuing and performing autopsies was invaluable to the investigation (4). These autopsies revealed pulmonary edema and large proteinaceous pleural effusions. At the first meeting of the three joint investigators (the New Mexico Department of Health, the Office of the Medical Investigator at the University of New Mexico School of Medicine, and CDC) a list was established of the most likely causes (i.e., influenza, plague, or a possible new agent) and intensive diagnostic efforts were mounted.

**FIGURE 1:**
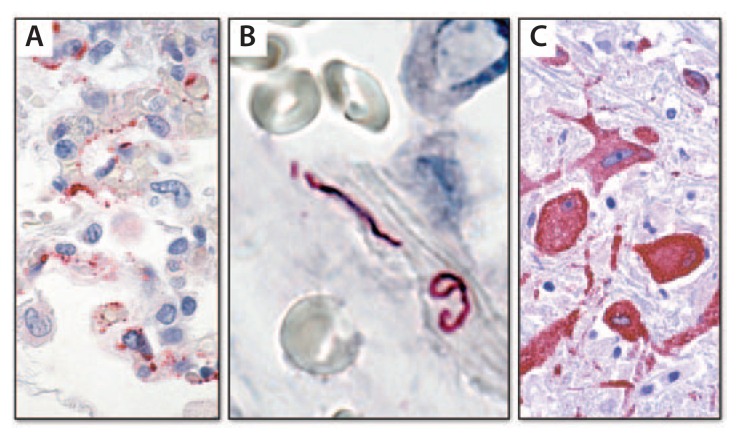
Immunohistochemistry for detecting pathogens in tissue* * Red color indicates site of the pathogens: A) Hantavirus proteins can be seen in endothelial cells in the lung of a patient; B) *Leptospira* organisms are present in large blood vessels in the lung; C) West Nile virus antigens can be seen in neurons in a patient with encephalitis.

The first breakthrough came via serologic testing at CDC with the detection of hantaviral antibodies in serum of patients who had succumbed to the illness (5). This was an unexpected finding because, at that time, there was no known pathogenic hantavirus in the United States, and all characterized pathogenic hantaviruses in other parts of the world caused renal disease with hemorrhage, unlike the pulmonary nonhemorrhagic disease observed in the Four Corners patients. Proof that this illness was caused by a hantavirus arrived rapidly through two hantavirus-specific tests developed at CDC. One test was a hantavirus-specific polymerase chain reaction (PCR) that was used to amplify the hantaviral nucleic acid sequence directly from the patient’s tissues and demonstrated that the infectious agent was a novel hantavirus (6). The other test was an immunohistochemical test using an antibody that reacted with all known hantaviruses. Using this antibody, microscopic examination of tissues from victims of this unexplained respiratory illness enabled localization of the viral proteins to the areas of disease in the lung, specifically the pulmonary endothelial cells (Figure 1A) (7). Immunohistochemistry (IHC) also provided a clue as to why patients developed “pulmonary leak”: the virus damaged pulmonary vessels very much like poking holes in a pipe.

At the outset of this investigation in 1993, only one nonpathogenic hantavirus had been identified in the United States; today, 24 hantaviruses with differing levels of pathogenicity have been identified in the Americas. This recognition of New World hantaviruses, coupled with a better understanding of hantavirus pulmonary syndrome, has resulted in critical improvements in the rapid recognition and clinical management of the disease and better understanding of the natural reservoir (rodents) and mode of transmission, all of which have greatly improved the ability to implement control and prevention measures, with emphasis on the critical role of individual communities.

### Leptospirosis in Nicaragua

In 1995, another pulmonary outbreak was reported, this time in Nicaragua, with several hundred cases and many deaths. An important difference with this outbreak was that instead of the clear fluid usually observed accumulating in the lungs, frank hemorrhage was detected (8). Initially, a viral hemorrhagic disease was suspected; however, within a few days pathologic evaluation helped solve the mystery. A novel IHC technique was used, employing several antibodies reactive against multiple strains of leptospirosis bacteria, and the etiology was confirmed (Figure 1B). The association of pulmonary hemorrhage with leptospirosis is now a well-recognized syndrome in addition to the classic hepatic and renal disease. This understanding, combined with awareness of increased transmission after intense rainfall and flooding and improved disease control and prevention efforts, resulted in better treatment, and ultimately saved lives.

### West Nile virus via transplantation

Transmission of infections from a single donor to multiple recipients through organ transplantation has been detected increasingly in recent years. Some infections identified at CDC as novel associations with solid organ transplants include West Nile virus (WNV), lymphocytic choriomeningitis virus, rabies, *Balamuthia*, and microsporidiosis (9–11). A young female victim of an automobile crash, whose care necessitated multiple transfusions, was associated with the first of these events in 2002 (12). Following her death, several organs were donated, and all recipients developed a febrile illness. One of the recipients who succumbed was thought to have contracted WNV infection, but results of his serology testing were negative for WNV. However, examination of autopsy specimens at CDC showed encephalitis, with IHC clearly demonstrating WNV antigen in neurons (Figure 1C), and the negative serology was determined to be a result of the transplant immunosuppression regimen. Diagnosis of this infection led to a traceback investigation that identified the blood components the donor had received prior to her death as the source of the virus and profoundly influenced thinking about West Nile virus transmission via blood transfusion and transplants.

## Autopsy-Based Surveillance Systems

Cause of death evaluation is an important component of the investigative process for emerging infectious diseases. When evaluating potentially infectious diseases as the causes of unexplained deaths, the use of autopsies has a number of advantages over death certificates: 1) availability of human tissues allows for enhanced diagnostic capacity and results in accurate determination of cause of death; 2) insights into pathogenesis and route of infection are gained; 3) rapid public health notification of findings is possible; and 4) recognition of additional infections not on death certificates is possible. The systematic collection and evaluation of this additional information affords an important opportunity for enhancing infectious disease surveillance. Monitoring of unexplained deaths and critical illnesses via autopsies at the state and local levels yields vital information about the actual numbers of cases of infectious diseases and provides insight into strategies for prevention.

### Med-X

The New Mexico Office of the Medical Investigator created a Medical Examiner Syndromic Surveillance System (Med-X) for all fatal infectious diseases, which can be used in medical examiner jurisdictions (13,14). A basic principle of the Med-X system is to seek organism-specific diagnoses in all potential infectious disease deaths investigated as unexplained by medical examiners. Designed initially to provide the capacity to identify fatalities resulting from bioterrorism and infections of public health importance, the model is based on two types of information: symptoms (Box) and pathologic syndromes found at autopsy (Table 2). The lists of both symptoms and syndromes are derived from most known bioterrorism-related illnesses. The symptom list (Box) serves to recognize and capture potential cases and drive decisions about autopsy performance; the syndrome list is used for early reporting of cases to the New Mexico Department of Health. For example, one of the 11 autopsy-based pathologic syndromes (community-acquired pneumonia and acute respiratory distress syndrome) might indicate the decedent had plague or tularemia; however, it is much more likely the decedent had influenza, pneumococcal disease, or various other more common conditions (Table 2). This information is valuable for public health officials in their decision-making regarding implementing prevention and control measures.

BOX **Symptoms tracked — Medical Examiner Syndromic Surveillance System (Med-X), New Mexico**

- Influenza-like symptoms
- Fever and respiratory symptoms
- Acute encephalopathy or new onset seizures
- Descending paralysis, polyneuropathy
- New fatal rash
- New jaundice
- Acute bloody diarrhea
- Unexpected death

In New Mexico during 2000–2002, a total of 6,104 medical examiner cases were examined. Of these, 250 met entry criteria (medical examiner autopsy case with a defined symptom or syndrome), of which 141 (56%) decedents had a target pathologic syndrome and 127 (51%) were found to have an infectious disease. Three symptom sets were found to be highly predictive of infection in an otherwise unexplained death: 1) fever and respiratory symptoms (72%), 2) influenza-like symptoms (65%), and 3) encephalopathy or new-onset seizures (50%); sudden unexpected death (19%) was found to be less likely to represent an infection. Furthermore, in 81% of infectious disease cases, an organism-specific diagnosis was determined, with 58% representing notifiable conditions in New Mexico, including *Streptococcus pneumoniae* (37 cases), *Streptococcus pyogenes* (eight cases), and *Haemophilus influenzae* (five cases), as well as one case each of *Mycobacterium tuberculosis* and botulism and two cases of human immunodeficiency virus (HIV) infection. These findings indicate the value of pathologists conducting routine microbiologic testing in cases that come under their jurisdiction and have symptoms predictive of infection.

### UNEX

**FIGURE 2:**
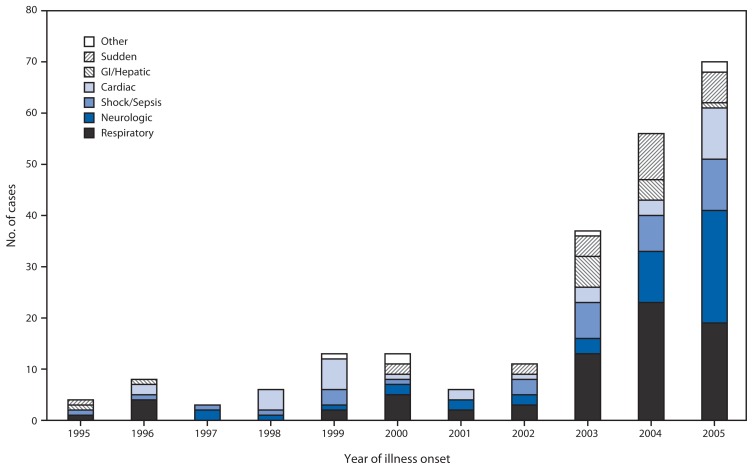
Unexplained deaths or critical Illnesses* — UNEX surveillance system, Minnesota, 1995–2005 **Abbreviations:** UNEX = Surveillance of Probable Infectious Etiology for Unexplained Death; GI = gastrointestinal. * In Minnesota, in addition to deaths, cases of unexplained critical illness also are included in the UNEX surveillance system. Cases in Minnesota include deaths or critical illnesses unexplained by routine testing that have premortem or postmortem findings suggestive of infectious etiology such as fever, leukocytosis, cerebrospinal fluid pleocytosis, or histopathologic evidence of an infection.

Another surveillance system using cause of death as a tool, Surveillance of Probable Infectious Etiology for Unexplained Death (UNEX), was initiated in 1995 as part of the CDC Emerging Infections Program in California, Connecticut, Minnesota, and Oregon. The goals of UNEX are to identify novel or newly emerging pathogens; to identify sudden, unexplained deaths attributed to known pathogens; to monitor the epidemiologic features of fatal infections; and to improve diagnostic postmortem testing (1). In Minnesota, cases of unexplained critical illness also are included in the UNEX surveillance system. Therefore, the cases include deaths or critical illnesses unexplained by routine testing that have premortem or postmortem findings suggestive of infectious etiology such as fever, leukocytosis, cerebrospinal fluid pleocytosis, or histopathologic evidence of an infection. Although persons who were previously healthy and aged <50 years are the focus of UNEX in Minnesota, the system is not limited to this population. UNEX cases are reported by clinical partners, including infectious disease physicians, infection preventionists, and hospital pathologists, whereas the main reporters outside of acute care facilities are medical examiners (1,15). Sources of information include autopsy and pathology reports, medical records, scene investigation findings, and biologic specimens; results are correlated with pathologic and clinical findings to determine the cause of death. During 1995–2005, respiratory cases were the most common syndrome in most years, with the number of these cases increasing over time (Figure 2).

### Med-X combined with UNEX

A Med-X surveillance system based on the New Mexico model also was initiated in Minnesota in 2006, enabling further description of infectious etiologies of death during 2006–2011. During this period, an average rate of 12 infectious deaths per 100,000 population was identified, encompassing 1,099 cases captured by UNEX and Med-X combined (723 UNEX cases, 908 Med-X cases, and 532 that fit the criteria for both systems). In all three groups, males predominated, and UNEX identified 70 critical illnesses and 228 deaths in persons aged <50 years who were previously healthy and had specimens available for testing (i.e., the UNEX subgroup). Overall, during 2006–2011, the etiology for 29% of cases that had a specimen available for testing was determined. Cases with a respiratory syndrome were most commonly explained and sepsis/shock was the next most commonly explained syndrome. Examining the method of diagnosis in the explained UNEX subgroup cases revealed that, whereas most pathogens were detected by PCR (including both pathogen-specific PCR and 16S-PCR), other techniques such as culture and IHC also were very useful.

Correlating laboratory findings, clinical features, and pathologic evidence to establish a causal relationship allows for the detection of organisms that otherwise would likely be missed. However, death investigation as a surveillance tool is not without its challenges. One hurdle commonly encountered was the identification of potential pathogens that are not the primary cause of a syndrome or death. Another obstacle was the resource-intensive nature of the surveillance and additional testing and materials required of medical examiners, pathologists, and public health staffs and laboratories.

## Conclusion

Effective use of basic and advanced diagnostic tools with ongoing development of new tools, a multidisciplinary approach, and vigilance by all critical partners are important in maintaining the partnership between pathology and public health. Tapping into the individual skills of clinicians, epidemiologists, microbiologists, veterinarians, pathologists, research scientists, and public health officials, especially in cases of unexplained deaths, contributes to the overarching goal of protecting the public from emerging infectious diseases and threats.

## Figures and Tables

**TABLE 1:** Emerging or newly recognized infections — worldwide, 1993–2004

Year	Disease	Country
1993	Hantavirus pulmonary syndrome	United States
1994	Plague	India
1995	Ebola hemorrhagic fever	Zaire
	Leptospirosis	Nicaragua
1996	New variant Creutzfeldt-Jakob disease	United Kingdom
1997	H5N1 influenza (avian)	Hong Kong
	Vancomycin-intermediate *Staphylococcus aureus*	Japan, United States
1998	Nipah virus encephalitis	Malaysia, Singapore
1999	West Nile encephalitis	Russia, United States
2000	Rift Valley fever	Kenya, Saudi Arabia, Yemen
	Ebola hemorrhagic fever	Uganda
2001	Foot and mouth disease	United Kingdom
	Anthrax	United States
2002	Vancomycin-resistant *Staphylococcus aureus*	United States
2003	Severe acute respiratory syndrome	Approximately 25 countries
	Monkeypox	Midwestern United States
2004	H5N1 influenza (avian)	Eight Asian countries

**TABLE 2:** Example of a pathology-based syndrome with linkage to potential bioterror illnesses and to illnesses that are more likely — Medical Examiner Syndromic Surveillance System (Med-X), New Mexico

Autopsy syndrome	Potential bioterror illness	More likely illness
Community-acquired pneumonia and acute respiratory distress syndrome	Plague	Influenza
	Tularemia	Pneumococcal and other bacterial and viral pneumonias
	Q fever	Hantavirus pulmonary syndrome
	Inhaled *Staphylococcus aureus*	
	Enterotoxin B	
	Ricin	
	Phosgene	
	Chlorine	
	Other gases	

## Footnotes

* Additional information available at http://www.onehealthinitiative.com.
